# Dissociation between systemic and pulmonary anti‐inflammatory effects of dexamethasone in humans

**DOI:** 10.1111/bcp.12857

**Published:** 2016-01-15

**Authors:** Johann Bartko, Leopold Stiebellehner, Ulla Derhaschnig, Christian Schoergenhofer, Michael Schwameis, Helmut Prosch, Bernd Jilma

**Affiliations:** ^1^ Department of Clinical PharmacologyMedical University of ViennaViennaAustria; ^2^ Department of PulmonologyMedical University of ViennaViennaAustria; ^3^ Department of Emergency MedicineMedical University of ViennaViennaAustria; ^4^ Department of RadiologyMedical University of ViennaViennaAustria

**Keywords:** acute respiratory distress syndrome, dexamethasone, lipopolysaccharide, lung inflammation, surfactant protein D

## Abstract

**Aims:**

The local pulmonary inflammatory response has a different temporal and qualitative profile compared with the systemic inflammatory response. Although glucocorticoids substantially downregulate the systemic release of acute‐phase mediators, it is not clear whether they have comparable inhibitory effects in the human lung compartment. Therefore, we compared the anti‐inflammatory effects of a pure glucocorticoid agonist, dexamethasone, on bronchoalveolar lavage and blood cytokine concentrations in response to bronchially instilled endotoxin.

**Methods:**

In this randomized, double‐blind and placebo‐controlled trial, 24 volunteers received dexamethasone or placebo and had endotoxin instilled into a lung segment and saline instilled into a contralateral segment, followed by bronchoalveolar lavage.

**Results:**

Bronchially instilled endotoxin induced a local and systemic inflammatory response. Dexamethasone strongly blunted the systemic interleukin (IL) 6 and C‐reactive protein release. In sharp contrast, dexamethasone left the local release of acute‐phase mediators in the lungs virtually unchanged: bronchoalveolar lavage levels of IL‐6 were only 18% lower and levels of IL‐8 were even higher with dexamethasone compared with placebo, although the differences between treatments were not statistically significant (*P* = 0.07 and *P* = 0.08, respectively). However, dexamethasone had inhibitory effects on pulmonary protein extravasation and neutrophil migration.

**Conclusions:**

The present study demonstrated a remarkable dissociation between the systemic anti‐inflammatory effects of glucocorticoids and its protective effects on capillary leak on the one hand and surprisingly low anti‐inflammatory effects in the lungs on the other.

## What is Already Known about this Subject

- Glucocorticoids are known to substantially downregulate the systemic inflammatory response. To date, it is unclear whether glucocorticoids have comparable inhibitory effects in the human lung. We therefore investigated the anti‐inflammatory effects of a pure glucocorticoid agonist, dexamethasone, on the local and systemic inflammatory response to bronchially instilled endotoxin.

## What this Study Adds

- Our findings demonstrated that the local release of acute‐phase mediators in the lungs is virtually unchanged in dexamethasone‐treated individuals, which is in sharp contrast to the strong systemic inhibitory effects of dexamethasone.
- This trial reports the strengths and limitations of systemic glucocorticoids on lung inflammation in response to endobronchial endotoxin.

## Introduction

To date, glucocorticoids have been widely used as anti‐inflammatory drugs in pulmonary medicine. Administered at pharmacological doses, glucocorticoid analogues suppress the inflammation and immune responses associated with pulmonary diseases such as asthma, organ rejection following transplantation, acute exacerbation of chronic obstructive pulmonary disease (COPD), pneumonia, acute respiratory distress syndrome (ARDS) and toxic pulmonary oedema. Central features of acute lung inflammation are the accumulation of neutrophils and a plasma exudate outside of blood vessels. The neutrophil recruitment is mediated through a strong gradient of chemokines and the extravasation of fluid is the result of an increase in the permeability of the pulmonary capillaries 1.

These early steps in lung inflammation are thought to be substantially downregulated by glucocorticoids. In rodents, the intratracheal administration of endotoxin, a component of Gram‐negative bacteria, is a commonly used lung inflammation model to test new therapeutic approaches 2, 3, 4. Therapeutic doses of synthetic glucocorticoids have been shown to inhibit pulmonary neutrophil influx and plasma protein leakage after intratracheal endotoxin administration in mice, accompanied by a substantial downregulation of inflammatory cytokine and chemokine production 5. Comparable inhibitory effects were reported in human volunteers challenged with endotoxin intravenously. Oral prednisolone inhibited the systemic release of inflammatory cytokines [tumour necrosis factor‐α (TNF‐α) and interleukin (IL) 6] and chemokines (IL‐8 and monocyte chemoattractant protein‐1) in a dose‐dependent manner 6. Interestingly, the alveolar space remains relatively insulated from high circulating levels of inflammatory cytokines during human endotoxaemia 7.

As the lung compartment is mostly spared and no sufficient data on this compartment are available, we hypothesized that the effects of glucocorticoids on the inflammatory response may differ in localized pulmonary endotoxin challenge from systemic endotoxin administration.

Therefore, we used a human model of local pulmonary inflammation based on direct segmental instillation of endotoxin developed by investigators from the National Institutes of Health (NIH) 8. In this model, endotoxin instillation is followed by saline instillation to the contralateral lung, which enables each individual to act as their own control and allows for comparison of a local response with that of the unchallenged lung and circulation. After endotoxin instillation, a rise in bronchoalveolar lavage (BAL) cellularity in endotoxin‐challenged samples, along with significant changes in pulmonary permeability, is seen. Moreover, proinflammatory mediators were increased substantially in the challenged lung segments 6 h after endotoxin instillation, most of them returning to basal levels by 24 h 8. Pulmonary endotoxin challenge is generally deemed as a safe and reliable experimental method for investigating the inflammatory response in healthy volunteers, or asthma or COPD patients 9, 10, 11. In the present study, the anti‐inflammatory and anti‐oedema effects of a pure glucocorticoid agonist, dexamethasone, on BAL and blood cytokine concentrations were compared in response to bronchially instilled endotoxin. We found a marked dissociation between the systemic and the local anti‐inflammatory effects of dexamethasone.

## Methods

### Study subjects, design and treatment

**Figure 1:**
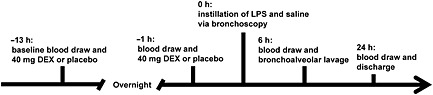
Schematic of the experimental design. DEX, dexamethasone; LPS, lipopolysaccharide

The ethics committee of the Medical University of Vienna approved the study protocol and the trial was conducted in accordance with the Declaration of Helsinki and registered at ClinicalTrials.gov as NCT01714427. Twenty‐four healthy nonsmokers gave written informed consent before study entry. Medical screening included medical history, physical examination, laboratory parameters, virology, chest radiography, spirometry and standard drug screening, and was unremarkable in all study participants. The trial was randomized, double blind and placebo controlled. Subjects (nine women, 15 men) were randomized into two groups – dexamethasone or placebo infusion – and additionally to instillation of 4 ng·kg^−1^ lipopolysaccharide (LPS) or saline into the left or right lungs. Volunteers received two separate doses of dexamethasone [40 mg in 100 ml saline (Merck, Vienna, Austria)] or placebo (physiological saline) intravenously on the first trial day 13 h prior to, and on the second trial day 1 h prior to endotoxin instillation (Figure 1). Dexamethasone is a commonly used synthetic glucocorticoid hormone with a 30‐fold higher anti‐inflammatory activity than hydrocortisone and no affinity for mineralocorticoid receptors 12. According to the summary of product characteristics, doses of 80–160 mg·day^−1^ are used for the treatment of noncardiogenic pulmonary oedema 13. Thus, given that its half‐life is168–324 minutes, two separate doses of 40 mg b.i.d. were deemed sufficient to reduce the lung inflammation response and putative pulmonary fluid accumulation. Endotoxin was prepared from national reference endotoxin (*Escherichia coli* O:113, CC‐RE‐Lot 3, NIH) by reconstitution with saline to 4 ng·kg^−1^ body weight in a total volume of 2 ml. A bilateral BAL was performed 6 h after endotoxin instillation. Volumes of 140 ml prewarmed saline (aliquots of 20–40 ml) were instilled into each lung site. The retrieved BAL volumes were comparable between LPS‐challenged and saline‐exposed segments [placebo: LPS 45 (35–50) ml *vs*. saline 54 (39–59) ml; dexamethasone: LPS 49 (43–64) ml *vs*. saline 53 (45–61) ml]. Vital signs (heart rate, continuous oxygen saturation and blood pressure) were monitored before the infusion of dexamethasone or placebo (13 h and 1 h before endotoxin instillation), at 20 min intervals after endotoxin instillation and for a minimum of 3 h after BAL; thereafter, subjects were allowed to leave the ward and then return the next morning for the 24‐h blood drawing, spirometry, vital sign measurements and physical examination. Blood samples were obtained at the screening visit, before drug administration (13 h and 1 h before endotoxin instillation), and 6 h and 24 h after endotoxin instillation.

### Assays and cellularity

Native BAL fluids were collected on ice and BAL leukocyte counts were immediately determined on an automated cell counter (XE‐5000, Sysmex Corporation, Kobe, Japan). BAL differential cell counts were done by morphological examination of the cytospin preparation (Shandon cytospin 3 centrifuge, Cheshire, UK) stained with modified Wright's stain. Lavage samples were than centrifuged and the supernatant was collected and stored at −80 °C until assays were performed. Blood and BAL concentrations of IL‐6, IL‐8, TNF‐α and surfactant protein D (SP‐D) were measured using specific enzyme immunoassays (R&D Systems, Minneapolis, MN, USA). The lower limits of quantification are 0.156 pg·ml^−1^ for IL‐6, 1.6 pg·ml^−1^ for IL‐8, 0.5 pg·ml^−1^ for TNF‐α and 0.63 ng·ml^−1^ for SP‐D, and allow the detection of even very low normal values in healthy volunteers 14, 15. C‐reactive protein (CRP), blood differential, BAL total protein and immunoglobulin (Ig) G were determined in an accredited routine laboratory.

### Statistical analysis

The primary comparison of interest was the difference in IL‐6 levels in BAL samples between treatment groups. Our sample size calculation was based on previous published results showing a coefficient of variation of 47% in peak IL‐6 levels in BAL samples 8. We calculated that 12 subjects per group would suffice to detect 60% lower IL‐6 levels in the dexamethasone group. Values are expressed as median and interquartile range (IQR) unless otherwise noted. A repeated measures analysis of variance was followed by nonparametric tests for reasons of non‐normal distribution of data. Statistical comparisons between groups were performed using the Mann–Whitney *U* test, and between lung sites of individuals using the Wilcoxon test. All statistical calculations were performed using commercially available statistical software (Statistica Version 6.1; Stat Soft, Tulsa, OK, USA).

## Results

**Figure 2:**
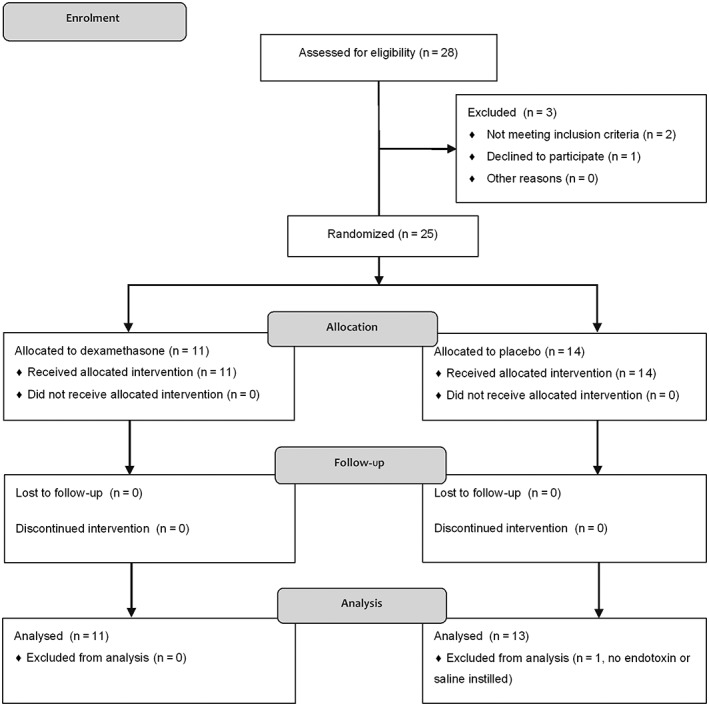
Consolidated Standards of Reporting Trials flow diagram. Twenty‐eight subjects were screened, and three were excluded (two had a cough and fever a week before the first trial day, and one individual declined to participate). In one subject allocated to the placebo group, no endotoxin or saline was instilled because obstructive sleep apnoea was suspected when sedation was initialized

From a total of 28 screened volunteers, three subjects were excluded. Two individuals had symptoms of a clinically relevant illness (cough and fever) a week before the first trial day, and one individual declined to participate. In one subject allocated to placebo, no endotoxin or saline was instilled because obstructive sleep apnoea was suspected when sedation was initialized and the subject was therefore excluded from analysis (Figure 2). Trial participants had comparable baseline characteristics (Table 1). The endotoxin challenge was well tolerated among all subjects and no severe adverse events occurred. Two subjects had a mild cough and one subject developed chills and fever transiently. Symptoms associated with the BAL procedure included fever (four subjects, all allocated to placebo; mean fever onset after BAL: 4.5 h), cough (eight subjects), throat pain (three subjects) and vomiting (two subjects). There was a small, but significant increase in body temperature, from a median of 35.9 °C to 36.3 °C (*P* = 0.012 at 7–9 h), which was slightly more pronounced among placebo‐treated individuals (median increase: placebo 0.45 °C *vs*. dexamethasone 0.30 °C).

**Table 1:** Characteristics of trial participants

	Placebo (*n* = 13)	Dexamethasone (*n* = 11)
**Female, n (%)**	6 (46)	3 (27)
**Age, years**	25.4 (3.7)	27.3 (3.8)
**BMI (kg·m^−2^)**	22.9 (2.5)	23.1 (3.1)
**FEV_1_, % predicted**	97.1 (7.1)	99.1 (6.7)

BMI, body mass index; FEV_1_, forced expired volume in 1 s. Values represent means (standard deviation) unless otherwise indicated.

### Bronchoalveolar inflammation in response to endotoxin

**Figure 3:**
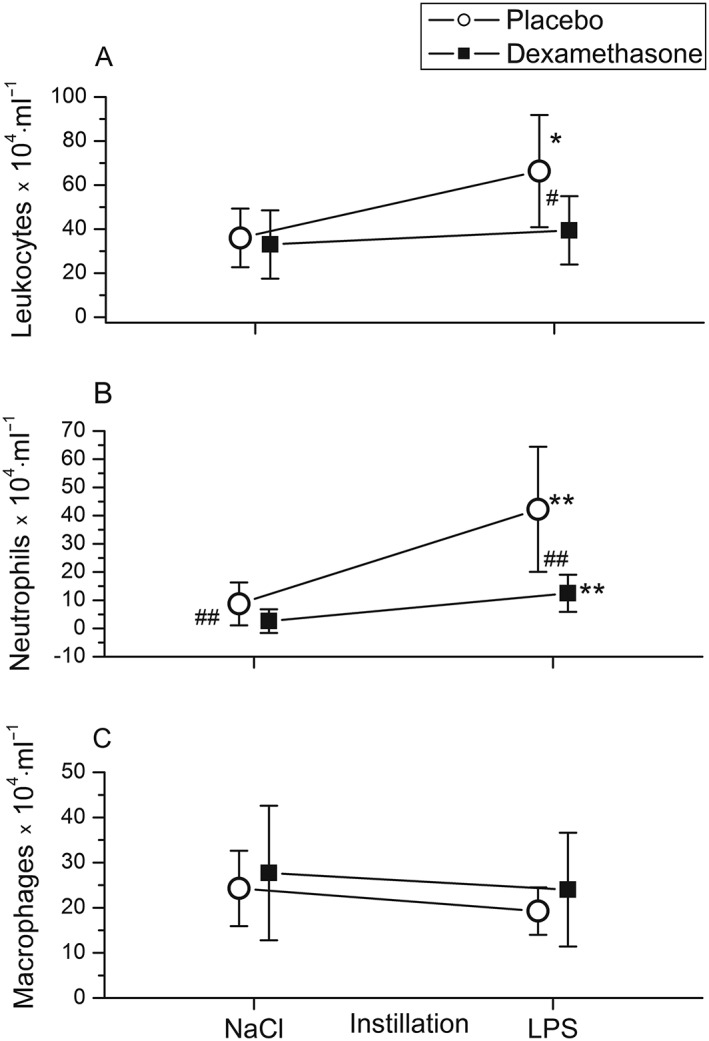
Instillation of 4 ng·kg^−1^ lipopolysaccharide (LPS) into a lung segment in healthy volunteers increased bronchoalveolar lavage (BAL) fluid leukocyte (A) and neutrophil (B) counts compared with BAL fluid from saline‐instilled (contralateral) lung sites. BAL was performed 6 h after pulmonary LPS instillation. Pretreatment with dexamethasone intravenously (■) (*n* = 11) inhibited the LPS‐induced rise in BAL fluid cellularity (A) and neutrophil counts (B) compared with placebo‐ treated (○)individuals (*n* = 13). BAL fluid concentrations of macrophages (C) were not altered significantly by LPS or dexamethasone. Symbols and lines represent means and 95% confidence intervals. *P < 0.05, **P < 0.01 *vs*. saline; ^#^ *P* < 0.05, ^##^ *P* < 0.01 for comparison between dexamethasone and placebo treatment

**Figure 4:**
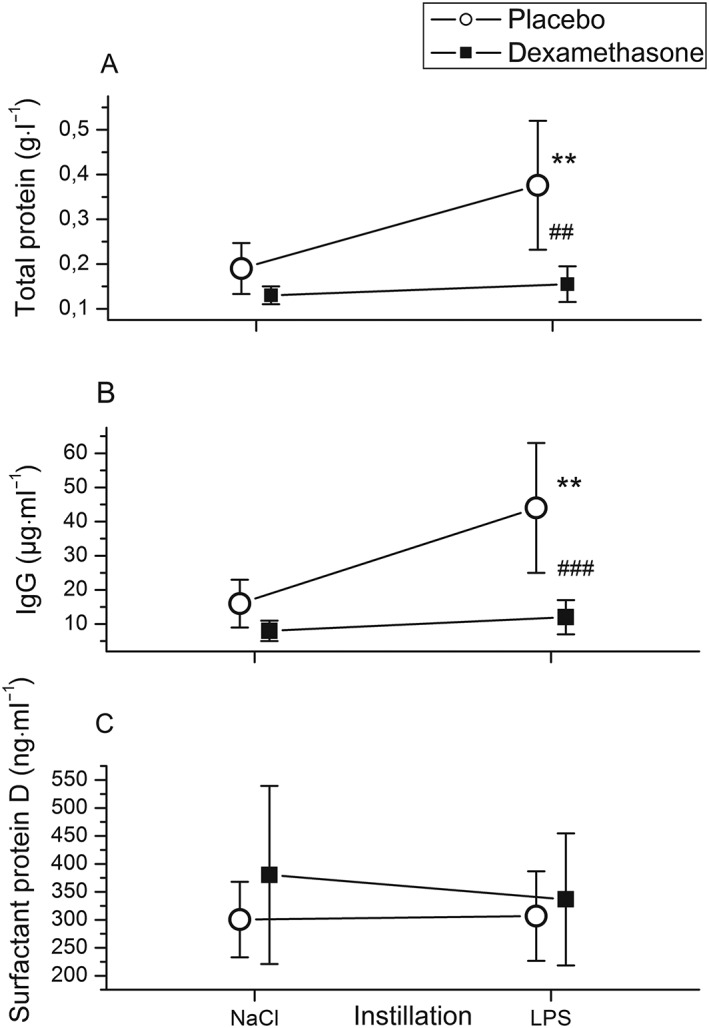
Instillation of 4 ng·kg^−1^ lipopolysaccharide (LPS) into a lung segment of healthy volunteers increased bronchoalveolar lavage (BAL) fluid total protein (A) and immunoglobulin G (IgG) (B) concentrations compared with BAL fluid from saline‐instilled (contralateral) lung sites. BAL was performed 6 h after pulmonary LPS instillation. Pretreatment with dexamethasone intravenously (■) (*n* = 11) inhibited the LPS‐induced rise in total protein (A) and IgG (B) concentrations compared with placebo‐treated (○) individuals (*n* = 13). BAL fluid concentrations of the epithelial lung injury marker surfactant protein D (C) were not altered by LPS or dexamethasone. Symbols and lines represent means and 95% confidence intervals. **P < 0.01 *vs*. saline; ^##^ *P* < 0.01, ^###^ *P* < 0.001 for comparison between dexamethasone and placebo treatment

**Figure 5:**
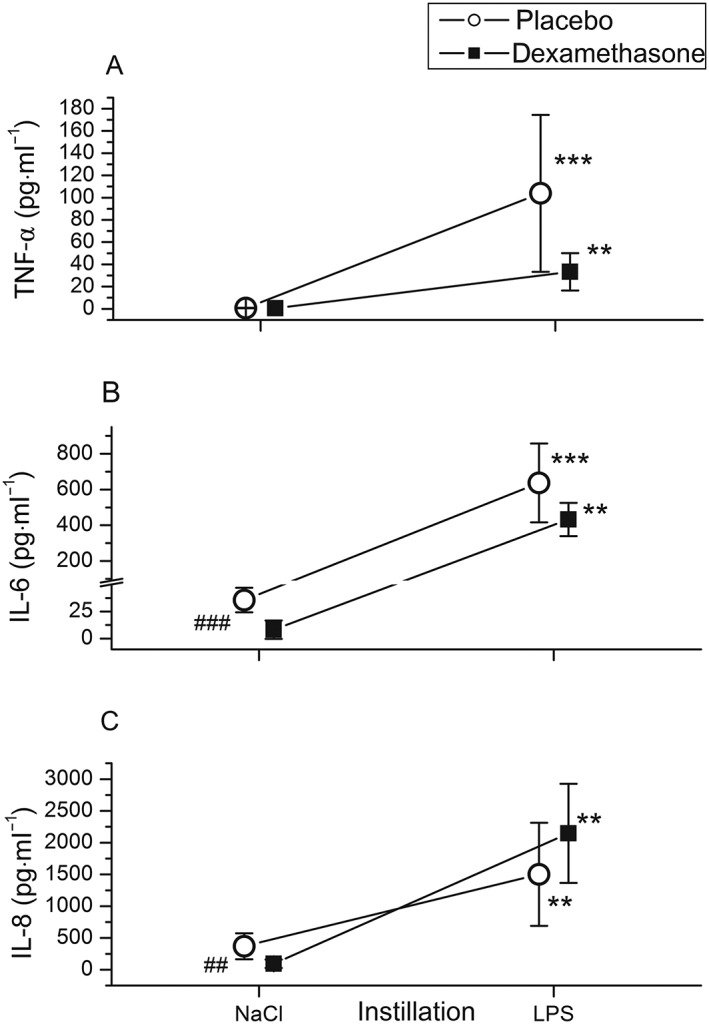
Cytokines in bronchoalveolar fluid 6 h after pulmonary instillation of 4 ng·kg^−1^ lipopolysaccharide (LPS) and saline into the contralateral lung from volunteers who received dexamethasone intravenously (■) (*n* = 11) or placebo (○) (*n* = 13). LPS strongly increased tumour necrosis factor‐α (TNF‐α) (A), interleukin (IL)‐6 (B) and IL‐8 levels (C). Dexamethasone gave rise to a relatively small decrease in IL‐6 (−18%) from LPS‐challenged segments in comparison with placebo (B) and failed to reduce TNF‐α (A) or IL‐8 (C) levels. Symbols and lines represent means and 95% confidence intervals. **P < 0.01, *** *P* ≤ 0.001 *vs*. saline. ^##^ *P* < 0.01, ^###^ *P* ≤ 0.001 for comparison between dexamethasone and placebo treatment

In endotoxin‐challenged segments, leukocyte counts increased by 80% (*P* = 0.028; Figure 3A), total protein by 50% (*P* = 0.011; Figure 4A) and IgG concentrations 2.4‐fold (*P* < 0.01; Figure 4B) in BAL fluid compared with BAL fluid from saline‐instilled segments. The increase in the leukocyte count was due to a tenfold rise in the neutrophil count (*P* < 0.001; Figure 3B), whereas macrophage (Figure 3C) and lymphocyte (data not shown) counts did not change significantly. Moreover, endotoxin increased BAL fluid TNF‐α levels 100‐fold, to 52 (22–129) pg·ml^−1^ (*P* < 0.001; Figure 5A). Endotoxin increased IL‐6 levels 13‐fold (*P* < 0.001; Figure 5B) and IL‐8 levels fivefold (*P* < 0.005; Figure 5C) in BAL fluid, compared with BAL fluid after saline instillation (Table 2).

**Table 2:** Bronchoalveolar lavage fluid characteristics

BAL fluid measures	Placebo (*n* = 13)		*P* value	Dexamethasone (*n* = 11)		*P* value
	Control	Endotoxin		Control	Endotoxin	
**WBC (×10^4^ cells·ml^−1^)**	26 (21–57)	47 (41–91)	0.028	23 (18–50)	27 (23–60)*	0.55
**PMN (×10^4^ cells·ml^−1^)**	3.1 (2.3–8.1)	29.7 (19.8–53.6)	<0.001	0.9 (0.3–1.3)‡	6.2 (5.2–19.1)†	0.003
**Protein (g·l^−1^)**	0.18 (0.12–0.22)	0.27 (0.25–0.47)	0.011	0.12 (0.1–0.16)	0.14 (0.11–0.2)†	1.000
**IgG (μg·ml^−1^)**	13 (8–20)	31 (26–62)	0.006	7 (4–11)*	9 (5–17)‡	0.343
**TNF‐α (pg·ml^−1^)**	0.5 (0.5–0.8)	52 (22–129)	<0.001	0.5 (0.5–0.5)	24.0 (19.5–45.5)	0.003
**IL‐6 (pg·ml^−1^)**	42 (25–49)	564 (487–725)	0.001	3.9 (1.8–8.5)‡	463 (323–507)	0.003
**IL‐8 (pg·ml^−1^)**	228 (122–464)	1152 (429–1780)	<0.005	58 (42–95)†	1968 (1458–2620)	0.003
**SP‐D (ng·ml^−1^)**	313 (219–398)	340 (184–403)	0.861	346 (293–412)	293 (247–423)	0.351

BAL, bronchoalveolar lavage; IgG, immunoglobulin G; IL, interleukin; PMN, polymorphonuclear neutrophils; SP‐D, surfactant protein D; TNF‐α, tumour necrosis factor‐α; WBC, white blood cells. Values represent medians (interquartile range). *P*‐values represent comparison between lung sites.

* *P* < 0.05 for comparison between dexamethasone and placebo treatment.

† *P* < 0.01 for comparison between dexamethasone and placebo treatment.

‡ *P* ≤ 0.001 for comparison between dexamethasone and placebo treatment.

### Effects of dexamethasone on pulmonary inflammation

Dexamethasone was a potent inhibitor of the endotoxin‐induced rise in: BAL fluid cellularity (*P* < 0.05; Figure 3A), neutrophil accumulation (*P* < 0.01; Figure 3B), and total protein (*P* = 0.003; Figure 4A) and IgG concentrations (*P* < 0.001; Figure 4B) compared with placebo (Table 2). By contrast, IL‐6 levels in BAL fluid from endotoxin‐challenged lung segments were only 18% lower with dexamethasone compared with placebo, yet the differences between treatments were not statistically significant (*P* = 0.07; Figure 5B) (Table 2). There was a trend toward higher IL‐8 and lower TNF‐α levels in BAL fluid with dexamethasone compared with placebo, but this also was not significant (*P* = 0.08 and *P* = 0.18, respectively) (Figure 5, Table 2).

### Effects of dexamethasone on saline‐challenged lungs

As placebo treatment is expected to be without effect, BAL samples from saline‐instilled lung segments may reflect conditions in the absence of inflammation with only minimal inflammatory mediator release and virtually natural levels of leukocytes.

IL‐6 and IL‐8 levels in BAL fluid from saline‐instilled lung segments were 90% (*P* = 0.001; Figure 5B) and 75% (*P* = 0.005; Figure 5C), respectively, lower with dexamethasone compared with placebo. Similarly, dexamethasone reduced neutrophil counts and BAL fluid IgG levels in saline challenged lung segments compared with placebo (*P* < 0.001 and *P* = 0.026, respectively; Figures 3B and Figure 4B). TNF‐α levels were low in BAL fluid from saline‐instilled lung segments after both placebo and dexamethasone infusion (0.5 pg·ml^−1^ in both groups; Figure 5A).

### Systemic inflammatory response after endotoxin instillation

**Figure 6:**
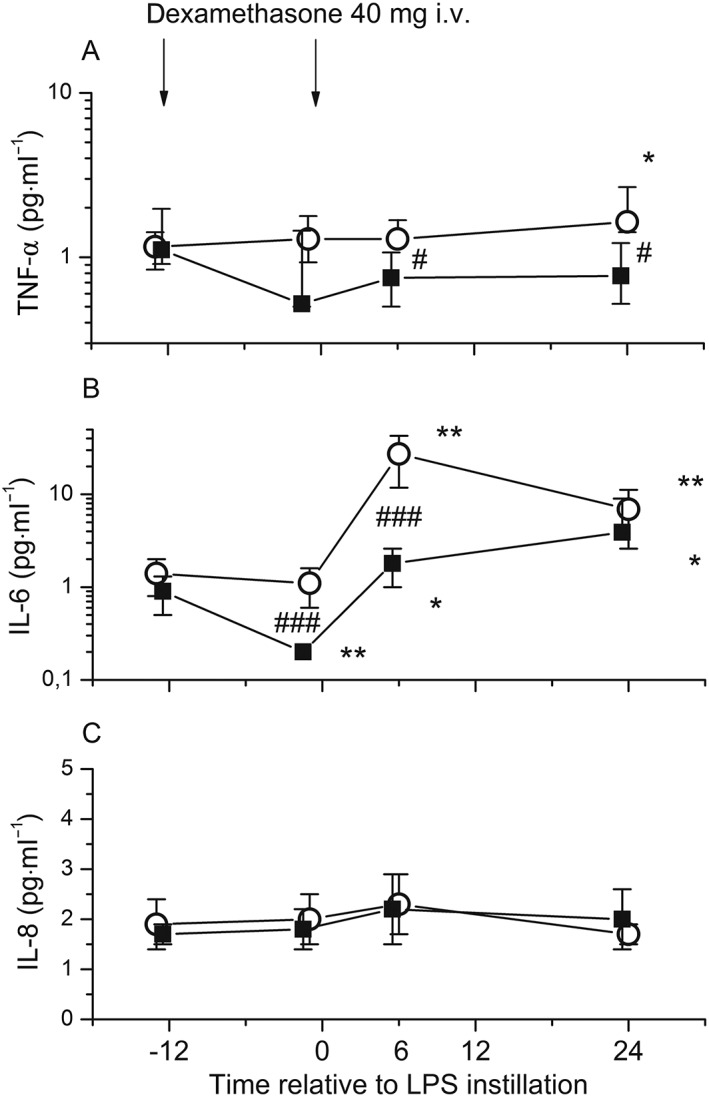
Systemic cytokine release in response to bronchial instillation of 4 ng·kg^−1^ lipopolysaccharide (LPS) in healthy volunteers who received dexamethasone intravenously (i.v.) (■) (*n* = 11) or placebo (○) (*n* = 13). Venous blood was obtained before drug administration (13 h and 1 h before LPS instillation), and 6 h and 24 h after LPS instillation. LPS instillation was associated with a minimal increase in TNF‐α levels (24 h) (A) and a significant increase in IL‐6 levels (*P* < 0.002, at 6 h) (B). IL‐8 (C) levels did not change over 24 h. Dexamethasone effectively reduced IL‐6 (B), whereas IL‐8 (C) remained unchanged and TNF‐α levels (A) were reduced moderately. In (A), data are displayed as median and interquartile range. In (B) and (C), data represent means and 95% confidence intervals. *P < 0.05, **P < 0.01 *vs*. baseline. ^#^ *P* < 0.05, ^###^ *P* < 0.001 for comparison between dexamethasone and placebo treatment

**Figure 7:**
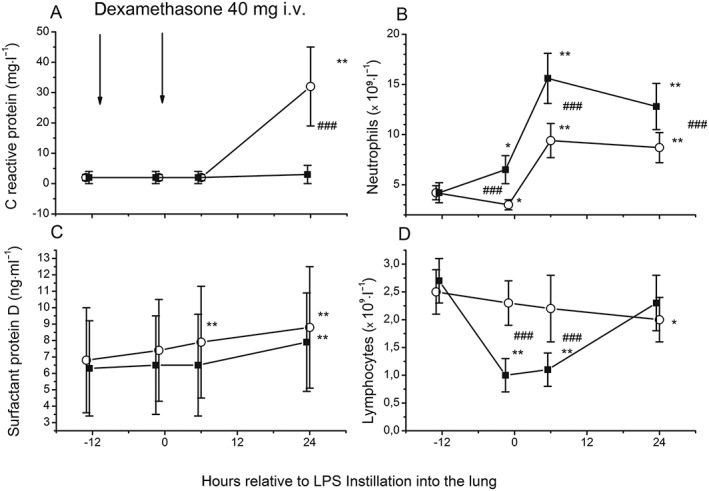
Changes in C‐reactive protein (A), absolute counts of neutrophils (B), plasma concentrations of surfactant protein D (C) and lymphocytes (D) in response to bronchial instillation of 4 ng·kg^−1^ lipopolysaccharide (LPS) in healthy volunteers who received dexamethasone intravenously (i.v.) (■) (*n* = 11) or placebo (○) (*n* = 13). Venous blood was obtained before drug administration (13 h and 1 h before LPS instillation), and 6 h and 24 h after LPS instillation. LPS instillation was associated with increases in C‐reactive protein and absolute neutrophil counts and a reduction of absolute lymphocyte counts. Dexamethasone almost completely inhibited the rise in C‐reactive protein, and this was accompanied by the expected lymphocytopenia and neutrophilia. Plasma concentrations of surfactant protein D rose significantly after endotoxin instillation in the placebo group (at 6 h and 24 h *vs*. baseline) and, less pronounced, in the dexamethasone group (at 24 h *vs*. baseline). Symbols and lines represent means and 95% confidence intervals. *P < 0.05, **P < 0.01 *vs*. baseline. ^###^ *P* < 0.005 for comparison between dexamethasone and placebo treatment

LPS instillation induced only a limited systemic inflammatory reaction. IL‐6 increased 22‐fold (6 h; *P* < 0.002; Figure 6B) and CRP increased 32‐fold (24 h; *P* < 0.002; Figure 7A), while plasma IL‐8 levels did not change over 24 h (Figure 6C) and TNF‐α increased only minimally (24 h; *P* = 0.01; Figure 6) (Table 3). Endotoxin instillation increased absolute neutrophil counts twofold (*P* = 0.002; Figure 7B) and reduced absolute lymphocyte counts by 30% (*P* = 0.016; Figure 7D) (Table 3).

**Table 3:** Systemic inflammatory response in peripheral blood

Blood measures	Placebo (*n* = 13)		Dexamethasone (*n* = 11)	
	Baseline	Maximum change (time)	Baseline	Maximum change (time)
**Neutrophils (× 10^9^cells·ml^−1^)**	4.1 (3.6–4.3)	9.5 (6.6–12.2) (6 h)†	4.3 (3.2–5.1)	14.7 (13.1–18.4) (6 h)†
**Lymphocytes (× 10^9^cells·ml^−1^)**	2.4 (1.9–2.7)	1.7 (1.6–2.6) (24 h)*	2.6 (2.1–3.2)	1.1 (0.8–1.6) (6 h)*
**CRP (mg·l^−1^)**	0.9 (0.2–1.6)	29 (12–52) (24 h)†	0.4 (0.2–3.6)	2 (0.2–4.1) (24 h)
**TNF‐α (pg·ml^−1^)**	1.2 (0.8–1.4)	1.6 (1.4–2.7) (24 h)*	1.1 (0.9–2.0)	0.8 (0.5–1.2) (24 h)*
**IL‐6 (pg·ml^−1^)**	0.8 (0.7–1.7)	17.7 (10.8–40.6) (6 h)†	0.9 (0.4–1.2)	1.8 (0.8–2.5) (6 h)*
**IL‐8 (pg·ml^−1^)**	1.6 (1.6–1.6)	1.6 (1.6–2.8) (6 h)	1.6 (1.6–1.6)	1.6 (1.6–2.3) (6 h)
**SP‐D (ng·ml^−1^)**	5 (2.9–8.9)	7.3 (4.6–11.3) (24 h)†	4.2 (2.8–8.8)	5.8 (4.5–11.7) (24 h)†

CRP, C‐reactive protein; IL, interleukin; SP‐D, surfactant protein D; TNF‐α, tumour necrosis factor‐α. Venous blood samples were drawn 13 h and 1 h before, and 6 h and 24 h after instillation of saline and endotoxin (4 ng·kg^−1^). Values represent medians (interquartile range).

* *P* < 0.05.

† *P* < 0.005.

### Effects of dexamethasone on the systemic inflammatory response

Dexamethasone reduced the systemic release of IL‐6 by 90% and blunted the rise in CRP (both *P* < 0.001 compared with placebo; Figures 6B and 7A). Similar to the placebo group, IL‐8 levels remained unchanged (Figure 6C). TNF‐α levels were moderately reduced after dexamethasone infusion compared with placebo (*P* < 0.05; at 6 h and 24 h; Figure 6A) (Table 3). Dexamethasone increased absolute neutrophil counts by about twofold compared with the placebo group, both before and after endotoxin instillation (*P* < 0.005 at –1 h, 6 h and 24 h; Figure 7B). This was accompanied by an approximately 50% decrease in absolute lymphocyte counts (*P* < 0.005 at −1 h and 6 h; Figure 7D) (Table 3).

### Effects of dexamethasone before endotoxin instillation

Plasma IL‐6 levels were suppressed by 80% (*P* < 0.001, Figure 6B) 12 h after the first dexamethasone infusion in comparison with placebo‐treated volunteers, whereas plasma TNF‐α, IL‐8 and CRP levels did not change significantly before endotoxin challenge (Figures 6A,C and 7A).

### SP‐D

The epithelial lung injury marker SP‐D was detectable in BAL fluid samples, but we did not observe an effect of dexamethasone or endotoxin on SP‐D concentrations in the BAL fluid (Table 2; Figure 4C). By contrast, plasma concentrations of SP‐D rose significantly after endotoxin instillation in the placebo group (at 6 h and 24 h; both *P* ≤ 0.003; Figure 7C) and in the dexamethasone group (at 24 h; *P* = 0.003; Figure 7C) (Table 3). In the dexamethasone group, the percentage change in SP‐D at 6 h after endotoxin challenge [2% (IQR 10% to 12% vs. baseline)] was moderate in comparison with that in the placebo group [21% (IQR 11% to 24%)] (*P* = 0.011; Figure 7C).

## Discussion

LPS, a major component of Gram‐negative bacteria, is a key mediator in the pathogenesis of acute lung inflammation 16. Instillation of LPS into a lung segment induces a self‐limited inflammatory process *in vivo* 8. The model shares several characteristics with the pathophysiological pathways observed in the early course of acute pulmonary inflammation 17. Considering the controversial role of glucocorticoids in inflammatory pulmonary diseases, we sought to characterize the effects of dexamethasone infusion on the LPS‐induced cytokine profile in the human lung.

Surprisingly, dexamethasone caused only mild inhibition of the endotoxin‐stimulated cytokine release into BAL fluid, although it profoundly reduced ‘constitutive’ cytokine release in saline‐challenged lung segments, and virtually abrogated systemic endotoxin‐induced IL‐6 and CRP responses. A particular strength of the current study was the placebo‐controlled, randomized design, using a well‐defined intrabronchial endotoxin instillation with concurrent control of the contralateral lung, permitting within‐subject comparisons of individuals' LPS‐induced cytokine response 8, 18.

Local lung inflammation increases vascular permeability; consequently, total protein and leukocytes accumulate into the alveolar space 19. In the current lung inflammation model, instillation of 4 ng·kg^−1^ endotoxin into a lung segment increased BAL neutrophil count, and total protein and IgG concentrations, indicating a leaky capillary endothelium. This was accompanied by a considerable increase in the levels of pro‐inflammatory cytokines TNF‐α, IL‐6 and IL‐8, which is in line with previous trials 8, 18.

Pretreatment with 2 × 40 mg dexamethasone almost completely inhibited the cellular response and the rise in total protein and IgG concentrations in BAL fluid. Thus, dexamethasone maintains the integrity of the endothelial–epithelial barrier during LPS‐induced lung inflammation, without affecting cytokine release. A reduction in BAL cellularity after dexamethasone treatment has been seen in most LPS‐based animal models, regardless of species 20, 21, 22. Similarly, dexamethasone was found to inhibit vascular leakage into the BAL fluid of mice 23.

In the current trial, dexamethasone gave rise to a relatively small decrease in IL‐6 levels (−18%) in the BAL fluid from endotoxin‐challenged segments in comparison with placebo‐treated individuals, and failed to reduce TNF‐α or IL‐8 levels in the BAL fluid of endotoxin‐challenged lung sites. By contrast, dexamethasone almost completely prevented LPS‐induced systemic inflammation. The rise in plasma IL‐6 levels after bronchial endotoxin challenge is largely caused by translocation from the pulmonary to the systemic compartment 24. Dexamethasone treatment has been shown to attenuate the IL‐6 gradient between right atrial and aortic blood after intratracheal endotoxin challenge in mice 25. This, together with the protective effects on the capillary leak we observed, suggests that dexamethasone has a compartmentalizing effect on the lungs.

The lack of cytokine suppression in the lung may have several causes. For example, recent investigations have indicated that LPS‐stimulated IL‐8 release from alveolar macrophages, the primary inflammatory cell in the lung compartment and a major source of IL‐8 and IL‐6 26, 27, is relatively insensitive to dexamethasone and that IL‐6 release is only partially suppressed 28, 29. A study investigating airway neutrophilia found that, in contrast to blood neutrophils, airway neutrophils have very low glucocorticoid receptor expression and, indeed, dexamethasone was found to suppress TNF‐α and IL‐8 release from sputum neutrophils to a much lesser extent than from those isolated from blood 30. Another well‐known effect of dexamethasone is the inhibition of neutrophil apoptosis 31; consequently, dexamethasone treatment could have primed neutrophils from the circulation to survive longer in the target tissue. Glucocorticoids are known to reduce neutrophil recruitment 32 and it is likely that dexamethasone had at least some inhibitory influence on the transvascular migration in our study, although there was a significant neutrophil influx in the LPS‐challenged segments among dexamethasone‐treated subjects. This, in conjunction with the low glucocorticoid receptor expression and the fact that neutrophils generate proinflammatory signals 1, may have contributed, albeit to a small degree, to the lack of cytokine suppression in the lung in the present inflammation model. Finally, dexamethasone's sensitivity/insensitivity is tissue specific, and factors such as intracellular glucocorticoid availability, hormone binding affinity, heat shock protein complexes and modulation of gene transcription may have played an additional role 33. Some evidence of the limited effect of glucocorticoids on airway inflammation *in vivo* arose from an LPS inhalation study, in which a 6‐day course of 20 mg prednisolone daily did not influence the levels of TNF‐α in sputum after LPS inhalation 34. Similarly, fluticasone propionate, a topical glucocorticoid used in COPD and asthma, had no effect on neutrophil or IL‐6 levels in sputum from healthy volunteers after LPS inhalation 35. Our data are in good agreement with clinical reports. In a small nonrandomized study, methylprednisolone treatment (~1 mg·kg^−1^ intravenously, given mainly for bronchial dilatation) was associated with lower systemic levels of IL‐6 and CRP, and reduced BAL fluid cellularity, but there was no decrease in IL‐6 levels in the BAL fluid of mechanically ventilated patients with severe pneumonia 36. Similarly to the present study, methylprednisolone was found to decrease systemic IL‐6 levels in early ARDS in a recent randomized trial 37.

In contrast to LPS‐challenged lung segments, dexamethasone suppressed IL‐6 levels by 90% and IL‐8 levels by 75% in the BAL fluid from saline‐challenged segments, which may indicate an inhibitory effect on ‘constitutive’ cytokine release in the lungs of healthy individuals. This is consistent with *in vitro* data showing a dexamethasone‐induced decrease in IL‐8 mRNA and protein levels by 60% under basal conditions in cultured alveolar macrophages. In contrast to the present study, dexamethasone pretreatment has also been found to reduce IL‐8 levels after LPS stimulation *in vitro* 38. Similarly to our saline‐challenged lung segments, another study found high‐dose methylprednisolone to cause a 60% reduction in IL‐6 levels, but no decrease in IL‐8 levels, in the BAL fluid, and to reduce plasma IL‐6 levels by ~80% after a relatively mild proinflammatory stimulus of pulmonary thromboendarterectomy 39. Hence, the anti‐inflammatory effects of glucocorticoids in the lung could be dependent on the severity of lung inflammation and/or the stimulus. Our finding that dexamethasone infusion decreased plasma IL‐6 levels by 80% after 12 h but before LPS or saline instillation, in comparison with the placebo group, indicates that dexamethasone has a substantial suppressant effect on systemic IL‐6 levels. Dexamethasone induced the expected lymphocytopenia and neutrophilia in the blood, both of which are well‐established effects of glucocorticoids 40.

SP‐D, a pulmonary protein produced by alveolar epithelial type 2 cells and Clara cells, plays an important role in the host defence against microbial lung infections 41. It is a consistent biomarker of direct ARDS 42, and plasma SP‐D levels have been associated with adverse outcomes in the acutely injured lung 43. In the present study, endotoxin instillation increased plasma SP‐D concentrations in the placebo group. This is in line with the results of a recent trial in healthy smokers, where inhalation of 30 μg LPS increased systemic SP‐D levels by 18% 44. It appears that small amounts of endotoxin instilled into a lung subsegment are sufficient to release SP‐D from the bronchoalveolar compartment into the circulation, which further supports SP‐D as a possible biomarker of lung inflammation. In the present study, dexamethasone suppressed the endotoxin‐induced rise in SP‐D, which is in agreement with a previous study showing a fall in serum SP‐D levels in COPD patients receiving oral prednisolone (20 mg·day^−1^) 45. The authors of the latter study proposed this could be due to reduced permeability resulting from glucocorticoid treatment. In contrast to the systemic SP‐D release, we found that local SP‐D concentrations in BAL fluid were not altered by endotoxin challenge or dexamethasone treatment. BAL fluid SP‐D levels do not rise until 24 h after intratracheal LPS challenge in mice 46. Thus, we cannot entirely exclude the possibility that BAL fluid SP‐D levels might be detectable in the current model from 6 h after segmental LPS challenge, but the further rise in systemic SP‐D levels is relatively minimal after 24 h.

The main finding of the present study, that high doses of glucocorticoids predominantly suppress LPS‐induced capillary leak formation but leave cytokine release in the lung largely unaffected, highlights the limitation of glucocorticoid therapy in the early course of lung inflammation. This may have clinical implications.

In situations where pulmonary infection with Gram‐negative bacteria is suspected, and where glucocorticoid treatment, for example, is used as an adjunct, clinicians should be aware of the limited effect of glucocorticoids on inflammatory cytokine release in the lung compartment, in spite of their major systemic anti‐inflammatory effects on the responses of such mediators as CRP. Interestingly, our data are consistent with the failure of high‐dose glucocorticoid to improve the outcome of ARDS 47, even though a continuous infusion of low‐dose methylprednisolone (1 mg·kg^−1^·day^−1^) may be beneficial 37, 48. Similarly, low‐dose steroid treatment might be more favourable than high doses in acute exacerbations of COPD, as proposed by a recent investigation 49. It would therefore be worthwhile to investigate if a lower dose of dexamethasone has comparable effects on capillary leak formation and pulmonary cytokine production in future trials. In addition, the failure of dexamethasone to inhibit cytokine release in the lung should prompt further research with drugs that affect specific mediators in this model or in clinical trials. Finally, we were able to confirm that, following LPS instillation, levels of IL‐6 in the blood increase earlier than those of CRP, which is consistent with previous LPS studies in healthy volunteers, regardless of LPS administration route 8, 50, 51, 52.

Some limitations of the present study should be addressed. The size of the inflammatory response induced by endotoxin is much smaller than that seen in patients with acute pulmonary inflammation, such as pneumonia or acute exacerbation in COPD. Lung inflammation in COPD is far more complex as it is a chronic disease and more common in older patients. The immune response of a patient is substantially influenced by comorbidities and thus the results of a study in healthy volunteers cannot be directly extrapolated to the clinical setting. Another limitation is that we limited the power to detect a lower level of TNF‐α or IL‐8 release in the dexamethasone group owing to the large variation (113%) in TNF‐α levels in the BAL fluid after endotoxin instillation.

In conclusion, the present study demonstrated a remarkable dissociation between the systemic anti‐inflammatory effects of glucocorticoids and protective effects on the capillary leak on the one hand, and the surprisingly low anti‐inflammatory effects in the lung compartment on the other.

## Competing Interests

All authors have completed the Unified Competing Interest form at http://www.icmje.org/coi_disclosure.pdf (available on request from the corresponding author) and declare no support from any organization for the submitted work, no financial relationships with any organizations that might have an interest in the submitted work in the previous 3 years and no other relationships or activities that could appear to have influenced the submitted work.

*We are indebted to Christa Drucker, Sabine Schranz, Karin Petroczi and Christa Firbas for their technical assistance. This research was supported by grant SFB54‐P04 of the Austrian Science Funds (FWF)*.

## Contributors

JB, BJ, HP and LS were responsible for the conception and design; JB, BJ, UD, MS, CS and HP were responsible for analysis and interpretation; JB, BJ, HP, LS, UD, MS and CS drafted the manuscript; JB, BJ, HP, LS, UD, MS and CS approved the final version of the manuscript.
